# Evaluation of Prelaminar Region and Lamina Cribrosa with Enhanced Depth Imaging Optical Coherence Tomography in Pseudoexfoliation Glaucoma

**DOI:** 10.4274/tjo.05882

**Published:** 2018-06-28

**Authors:** Mehmet Giray Ersöz, Duygu Kunak Mart, Leyla Hazar, Emre Ayıntap, İrfan Botan Güneş, Hakkı Özgür Konya

**Affiliations:** 1 İstanbul Retina Institute, Ophthalmology Clinic, İstanbul, Turkey; 2 University of Health Sciences, İzmir Tepecik Training and Research Hospital, Ophthalmology Clinic, İzmir, Turkey; 3 Kızıltepe State Hospital, Ophthalmology Clinic, Mardin, Turkey; 4 Borçka State Hospital, Ophthalmology Clinic, Artvin, Turkey; 5 Kemalpaşa State Hospital, Ophthalmology Clinic, İzmir, Turkey

**Keywords:** Enhanced depth imaging, lamina cribrosa, optical coherence tomography, prelaminar tissue, pseudoexfoliation glaucoma

## Abstract

**Objectives:**

To analyze optic nerve head images of pseudoexfoliative glaucoma (PXG) patients and healthy volunteers obtained with enhanced depth imaging spectral domain-optical coherence tomography (SD-OCT).

**Materials and Methods:**

Seventy patients with PXG and 68 age- and gender-matched healthy subjects were included in this prospective study. The prelaminar tissue and lamina cribrosa were imaged using spectralis OCT with the enhanced depth imaging technique. PXG disease stage was determined with visual field to evaluate relationships between prelaminar tissue thickness (PTT), lamina cribrosa thickness (LT) and disease severity.

**Results:**

There was no significant difference between the PXG group and control group with regard to age, gender, central corneal thickness, or axial length. The mean PTT (93.1±44.5 μm, p<0.05) and LT (206.3±33.6 μm p<0.05) values of the PXG group were significantly lower compared to the control group in enhanced depth imaging OCT measurements. The PXG patients were divided into stages according to visual field defect severity. While a significant difference was not detected in PTT based on disease stage (p>0.05), a statistically significant difference was detected between stages for LT (p<0.05).

**Conclusion:**

A thinner PTT was correlated with the presence of PXG but not with the severity of glaucoma. In addition, LT has a stronger relationship with disease severity and progression compared to PTT.

## Introduction

Pseudoexfoliation syndrome (PEX) is an age-related generalized basal membrane disease. It is characterized by the excessive and progressive accumulation of fibrillary material in various ocular and extraocular tissues.^1^ Pseudoexfoliative glaucoma (PXG) develops in the vast majority of patients with PEX.^2^ PXG is the most common form of secondary open angle glaucoma types.^3^ It is characterized by high intraocular pressure (IOP), severe fluctuation of IOP, rapid progression, poor prognosis.^4^ The structural changes in lamina cribrosa (LC) beside elevated IOP and severe fluctuation were suggested to be associated with poor prognosis.^5,6,7^ Elastotic changes were detected in the LC of eyes with PXG.^7^ In a study conducted with atomic force microscopy, the stiffness of the LC was reported to decrease in pseudoexfoliative eyes.^8^ In addition, LC deformation may lead to ischemia through the compressive effect on the laminar capillary.^9,10^ Since laminar region has been considered the primary site of axonal injury in glaucoma, these alterations may contribute to the rapid progression of PXG.^3^ The prelaminar region which covers the LC is composed of retinal ganglion cells, axon bundles, astrocytes, capillaries and extraocular material. Its thickness may reduce as the result of ischemia.^11^ Prelaminar tissue thickness (PTT) was also shown to decrease as a response to acute^12^ and chronic^13^ IOP elevation.

Although optical coherence tomography (OCT) can visualize the anterior margins of LC and prelaminar tissue, it cannot visualize the posterior margins of the LC. It is possible to safely visualize the posterior margins of the LC and optic nerve head (ONH) with enhanced depth imaging (EDI), which is present in spectral domain (SD)-OCT.^14,15,16,17^

In our study, we analyzed ONH images of PXG patients and healthy volunteers obtained with EDI SD-OCT. We aimed to investigate the presence of a significant difference between the PXG group and control group with regard to PTT and LC thickness (LT). We also planned to investigate the relationship between PTT, LC and PXG disease stage determined with visual field, retinal nerve fiber layer (RNFL) thickness and vertical cup/disc ratio.

## Materials and Methods

This prospective study was approved by the Medical Ethics Committee of İzmir Tepecik Training and Research Hospital. The study was carried out in adherence to the tenets of the Declaration of Helsinki and written informed consent was obtained from all participants. Seventy patients with PXG and 68 age- and gender-matched healthy subjects were recruited from October 2014 to May 2015. Medical histories and demographic data of all participants were noted. All subjects underwent ophthalmic examination including best-corrected visual acuity, central corneal thickness with non-contact specular microscope (sp-2000p, Topcon, Japan), axial length (Lenstar LS900, Haag-Streit AG, Koeniz, Switzerland), Goldmann applanation tonometry, slit-lamp biomicroscopy, gonioscopy, dilated fundus photography, visual field test with Octopus 101 automated perimetry (Interzeag AG, Schlieren, Switzerland) using G2 program (central 30-2 threshold strategy) and SD-OCT scanning (Spectralis OCT, Heidelberg Engineering, Heidelberg, Germany). The vertical cup/disc ratio was noted on dilated fundus examination. 

PXG was diagnosed when baseline IOP >21 mmHg, open anterior chamber, glaucomatous optic neuropathy, visual field defects typical of glaucoma and pseudoexfoliation material on the anterior lens capsule, pupillary margin, or both. The Hodapp-Anderson-Parrish system modified for Octopus perimetry was used to classify patients with glaucoma.^18^ PXG patients were stratified into five groups according to the severity of visual field defects. Stage 1 (early) glaucoma was characterized by a mean deviation score (MDS) of -0.7 to +4.4 dB; stage 2 (moderate) glaucoma by an MDS of +4.5 to +9.4 dB; stage 3 (advanced) glaucoma by an MDS of +9.5 to +15.3 dB; stage 4 (severe) glaucoma by an MDS of +15.4 to +23.1 dB and stage 5 (end-stage) glaucoma by an MDS of ≥ +23.2. 

The inclusion criteria for eyes were a best-corrected visual acuity of 20/40 or better, spherical refraction within ±5.0 diopters and cylinder correction within ±3 diopters. At least two visual field tests were performed to minimize the learning effect. Only reliable (false positive/negative under 15% and reliability factor under 15) and compatible visual field results were included. The control group participants had normal eye exam and perimetry. The exclusion criteria included cardiovascular disease, diabetes, head trauma, Alzheimer’s disease, history of stroke, claustrophobia, ocular trauma and other ocular disease affecting visual field and RNFL. Patients whose IOP could not be controlled with medical treatment and end-stage patients were excluded. If both eyes had PXG, one eye was randomly selected for inclusion in the study. 

### Peripapillary RNFL Measurement with Spectral Domain-Optical Coherence Tomography

All OCT assessments involved in the study were performed by the same experienced ophthalmologist. For OCT examination, the RNFL thicknesses were assessed by scanning a peripapillary circle with a diameter of 3.4 mm and 768 A-scans. Only well-centered images with a signal strength of >20 dB were used. The RNFL thicknesses were automatically segmented and measured using Spectralis software version 5.3.3.0.

### Measurement of Prelaminar Tissue Thickness and Lamina Cribrosa Thickness by Spectral Domain-Optical Coherence Tomography Enhanced Depth Imaging

**Figure 1:**
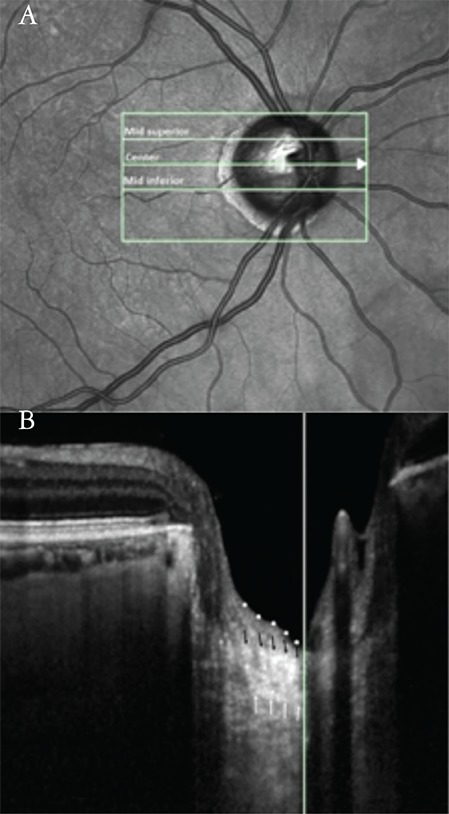
Simultaneous images of a pseudoexfoliation glaucoma patient. a) The measurement of prelaminar tissue thickness and laminar thickness (LT) were performed at the presumed vertical center of each of the 3 B-scans (mid-superior, center, mid-inferior). The short vertical line crossing the center horizontal line corresponds to the long white vertical line in the next image. B) The image shows a horizontal cross-sectional B-scan of the optic nerve head at the center line. The vertical white line marks the vertical center of the optic nerve head. The borders of the highly reflective region were accepted as the borders of the lamina cribrosa (LC); white arrows indicate the posterior borders and black arrows indicate the anterior borders of the LC. LT was defined as the distance between the anterior and posterior LC borders. Prelaminar tissue was defined as the reflective field on the anterior margin of the LC. White dots delineate the anterior borders of the prelaminar tissue

The prelaminar tissue and LC were imaged using the Spectralis OCT with the EDI technique. An internal nasal fixation light was used to center the disc in the 10´15° rectangle. This rectangle was scanned with 97 sections (384 A-scans) with an interval of 30 µm. An average of 45 frames was produced for each cross-sectional B-scan. Thickness measurements were done using Spectralis software version 5.3.3.0. LT and PTT were measured at the vertical center of ONH of 3 B-scans (mid-superior, center, mid-inferior). The center of the ONH was identified as the point where central retinal vessels originate from the ONH. Mid-superior and mid-inferior locations were determined as the midpoints between the center and the margins of the optic disc (Figure 1A). LT was defined as the distance between the anterior and posterior borders of the LC. The borders of the LC were considered to be where the highly reflective region started and finished. Prelaminar tissue was defined as the reflective field on the anterior margin of the LC (Figure 1B). For each patient, the mean of the measurements at the mid-superior, center and mid-inferior locations were regarded as the average PTT and LT. The average PTT and LT were used for statistical analyses. The relationship between PTT, LC and PXG disease stage was determined with visual field, RNFL thickness and vertical cup/disc ratio.

### Statistical Analysis

All statistical analyses were performed with Statistical Packages for the Social Sciences (SPSS, version 22, IBM corp., Armonk, New York, USA). A p value <0.05 was considered statistically significant. For comparison of groups, independent t test was used for continuous variables and chi-square test was used for categorical data. Comparison of the patients in different disease stages in the PXG group was done with Kruskal-Wallis test. Pearson correlation analysis was used for correlation analysis.

## Results

ONH EDI OCT images of 70 PXG patients and 68 healthy volunteers were analyzed. Two patients in the PXG group and 3 patients in the control group were excluded from the study because the posterior margins of the LC were not visualized clearly. Sixty-eight patients in the PXG group and 65 patients in the control group were included in the statistical analysis.

There was no significant difference between PXG group and control group with regard to age, gender, central corneal thickness, or axial length. Baseline characteristics of the participants are shown in Table 1.

Mean PTT (p<0.05) and LT (p<0.05) values of the PXG group were seen to be statistically significantly lower compared to the control group in EDI OCT measurements. While mean PTT was 93.1±44.5 µm in the PXG group, it was 213.9±141.1 µm in the control group. Mean LT was calculated as 206.3±33.6 µm in the PXG group and 269.1±24.1 µm in the control group.

The PXG patients were divided into stages according to visual field defect severity. There were 16 patients (23.5%) in early stage, 21 patients (30.9%) in moderate stage, 18 patients (26.5%) in advanced stage and 13 patients (19.1%) in severe stage. While a significant difference was not detected in PTT in comparison of disease stages (p>0.05), a statistically significant difference was detected between stages for LT (p<0.05). Post hoc multiple comparison results for LT are shown in Table 2.

**Figure 2:**
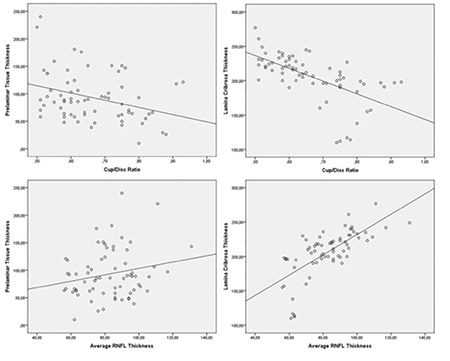
Scatter plots to exhibit correlations between enhanced depth imaging optical coherence tomography measurements vs. vertical cup/disc ratio and average retinal nerve fiber layer (RNFL) thickness

While a weak correlation was detected with vertical cup/disc ratio in correlation analysis done for PTT, a correlation was not detected with average of RNFL thicknesses (RNFLav). LT was found to be negatively correlated with vertical cup/disc ratio, positively correlated with RNFLav (Table 3, Figure 2).

## Discussion

The development of EDI in SD-OCT enabled clear visualization of prelaminar and laminar tissues and accelerated investigation of the relationships between these structures and glaucoma.^14,15,16,17,19^ Park et al.^16^ pointed out a limitation; with EDI OCT, the deeper portion and posterior border of the LC lack the clarity required for precise characterization of the structure. Recently, high-penetration OCT, also known as swept-source-OCT, which uses a center wavelength of approximately 1,050 nm instead of 840 nm (the wavelength used by current SD-OCT instruments), allows the imaging of deeper ocular layers, including the choroid and LC. It has been promised to enable more accurate characterization of the LC.^20,21^ In our study, patients whose posterior LC margins were not visualized clearly were excluded from the study. In the studies done using EDI, PTT was shown to decrease with the elevation of IOP and increased again following treatment.^12,13,22,23,24^ In addition, Jung et al.^13^ reported that prelaminar tissue is thinner in primary open angle glaucoma (POAG) patients compared to normotensive glaucoma patients (NTG). Chung et al.^25^ found PTT and LT low in progressing glaucoma patients compared to glaucoma patients who do not show progression. In the study of Chung et al.^25^, PTT and LT were found to be related to glaucoma progression; however, only LT was seen to be related to glaucoma progression in multivariate analysis.

To the best of our knowledge, this is the first study to investigate the relationship between PTT and PXG. In our study, PTT was significantly thinner in PXG patients whose IOP was within normal ranges with medical therapy compared to the control group. However, there was no significant difference between stages in the PXG group. In addition, PTT was poorly correlated with vertical cup/disc ratio and a correlation was not found with RNFLav. The standard deviation of PTT is high, which indicates that PTT values are distributed over a wide range in this patient group. The PTT values are also widely distributed in the population and are not homogeneously distributed. Therefore, there is no need to evaluate PTT in follow-up. The results of our study showed that a thinner PTT was correlated with the presence of PXG but not with the severity of glaucoma.

LC is one of the ocular structures where pathologic changes are seen in PEX syndrome.^5,6,7,26,27,28^ Insufficient LOXL1 tissue levels may lead to elastotic changes in affected tissues like LC.^29^ Braunsmann et al.^8^ reported that LC stiffness significantly decreased in their study done with cadaver eyes with PXG. Since the LC is the primary site of axonal injury in glaucoma, elastotic alteration and decreased LC stiffness may predispose to glaucoma development in patients with PEX.^3^ In a study performed with an SD-OCT EDI system, Park et al.^14^ found LT was significantly thinner in POAG and NTG patients compared to a control group. In addition, they showed that LT decreased as disease stage increased in glaucoma patients.^14^ Kim et al.^30^ reported that LC was thinner in PXG patients in similar disease stages compared to POAG patients. In our study, LT was lower in PXG patients compared to the control group. Mean LT was reported to decrease with the increase in disease stage. Park and Park^31^ determined that the diagnostic ability of LT is similar to peripapillary RNLF thickness and better than peripapillary RNFL thickness in early stage patients. Lee et al.^32^ reported that thin LC was associated with progressive RNLF thinning. In our study, LT was seen to be negatively correlated with vertical cup/disc ratio and positively correlated with RNFLav. In light of these data, it was concluded that LT may be a risk factor for the development of PXG. In addition, the laminar region could be one of the targets of glaucomatous injury. Long-term studies with more patients are needed to support this conclusion. 

### Study Limitation

A limitation of our study is that patients with PEX were not included; we only compared PXG patients and healthy subjects.

## Conclusion

In conclusion, thinning in PTT and LT parameters in SD-OCT EDI systems was correlated with the presence of PXG. In addition, LT has a stronger relationship with disease severity and progression compared to PTT. SD-OCT EDI mode is a recently developed technology and is not available in many centers and LT is not routinely assessed in glaucoma clinics. With advances in OCT systems, LT may be used for diagnosis and follow-up.

## Figures and Tables

**Table 1:**
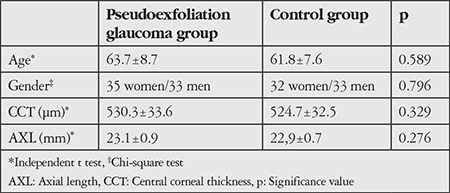
Baseline characteristics of the pseudoexfoliation glaucoma and control groups

**Table 2:**
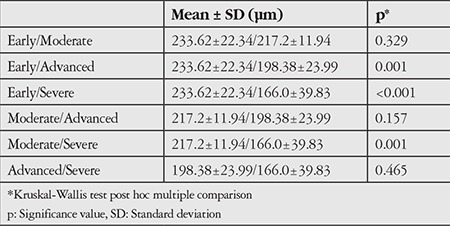
Multiple comparison of lamina cribrosa thickness of each group

**Table 3:**
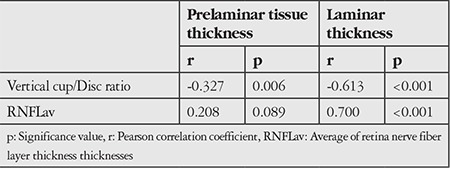
Correlation between enhanced depth imaging optical coherence tomography measurements vs. vertical cup/disc ratio and average retina nerve fiber layer thickness
